# Metformin and Glaucoma—Review of Anti-Fibrotic Processes and Bioenergetics

**DOI:** 10.3390/cells10082131

**Published:** 2021-08-19

**Authors:** Daire J. Hurley, Mustapha Irnaten, Colm O’Brien

**Affiliations:** 1 Department of Ophthalmology, Mater Misericordiae University Hospital, Eccles Street, D07 R2WY Dublin, Ireland; mirnaten2014@gmail.com (M.I.); cobrien@mater.ie (C.O.); 2 School of Medicine, University College Dublin, Belfield, D04 V1W8 Dublin, Ireland

**Keywords:** glaucoma, metformin, lamina cribrosa, fibrosis, AMPK, TGF-β, bioenergetics, mitochondrial function, signalling pathways, NAD

## Abstract

Glaucoma is the leading cause of irreversible blindness globally. With an aging population, disease incidence will rise with an enormous societal and economic burden. The treatment strategy revolves around targeting intraocular pressure, the principle modifiable risk factor, to slow progression of disease. However, there is a clear unmet clinical need to find a novel therapeutic approach that targets and halts the retinal ganglion cell (RGC) degeneration that occurs with fibrosis. RGCs are highly sensitive to metabolic fluctuations as a result of multiple stressors and thus their viability depends on healthy mitochondrial functioning. Metformin, known for its use in type 2 diabetes, has come to the forefront of medical research in multiple organ systems. Its use was recently associated with a 25% reduced risk of glaucoma in a large population study. Here, we discuss its application to glaucoma therapy, highlighting its effect on fibrotic signalling pathways, mitochondrial bioenergetics and NAD oxidation.

## 1. Introduction

Glaucoma is the leading cause of irreversible blindness worldwide, affecting more than 70 million people, with primary open angle glaucoma (POAG) accounting for 74% of cases [1,2]. It is more common in the older population, with a global prevalence of 3.5% in people over 40 years old and an adjusted odds ratio of 1.73 per decade age increment [3]. Glaucoma prevalence is projected to increase to over 110 million by the year 2040 as a result of longer life expectancy and a subsequently aging population [3].

Whilst age is the most commonly associated risk factor for POAG, it is of course non-modifiable [4,5,6]. Therefore, current glaucoma treatment paradigms revolve around targeting the main modifiable risk factor, intraocular pressure (IOP) [7]. Whilst lowering IOP has proven to be an effective measure in managing glaucoma, progression still occurs in over half of patients with optimal medical and surgical management, albeit at a slower rate [8,9]. Many patients are unable to achieve adequate target pressures or tolerate the IOP-lowering medications [10,11]. Furthermore, normal tension glaucoma (NTG), in which IOP lowering regimens are less efficacious, accounts for a large proportion of glaucoma cases, between 30–39% of Caucasian glaucoma cases and 52–92% of Asian glaucoma cases [12,13]. Thus, exists an unmet clinical and public health need to develop a disease modifying agent to prevent or further slow the progression of glaucoma.

Metformin, a biguanide derivate commonly used in patients with type 2 diabetes, has come to be a front-runner recently in addressing this need, following a line of publications linking its use in a diabetic population with reduced incidence of POAG [14,15]. The cellular mechanism through which metformin is acting to combat glaucoma is not yet understood but likely involves effects on the pathogenesis of fibrosis and mitochondrial bioenergetic dysfunction in glaucoma. This review aims to identify and examine some of these processes and postulate mechanisms through which metformin may be of vital use as a treatment adjunct in glaucoma in the wider population.

## 2. Primary Open Angle Glaucoma

POAG is an chronic, multifactorial optic neuropathy characterized by progressive loss of retinal ganglion cell (RGC) axons resulting in gradual loss of peripheral vision [1,16]. The trabecular meshwork (TM), a fenestrated structure located within the iridocorneal angle, is the main pathway for aqueous humour drainage and its dysfunction is largely responsible for IOP elevation [17,18]. The lamina cribrosa (LC), a mesh-like connective tissue structure at optic nerve head through which unmyelinated RGC axons pass as they exit the eye, is a key site of RGC axonal injury in POAG [19,20]. Abnormally elevated intraocular pressure results in mechanical distortion and stretching of RGC axons at the LC, thus impeding axoplasmic transport, reducing ocular perfusion and leading to visual field loss [21,22,23,24]. This involves several pathological features such as cupping of the optic nerve head, excessive accumulation of extracellular matrix (ECM) in the LC and TM and upregulation of pro-fibrotic cytokine transforming growth factor β (TGF-β) and extracellular matrix genes, collagen 1α1 (COL1α1) and α-smooth muscle actin (α-SMA) [25,26,27,28,29].

### 2.1. Fibrosis

Fibrosis is the final common pathway of most chronic inflammatory conditions and is defined by the accumulation of excess ECM components leading to pathological tissue destruction and impaired organ function [30]. Fibrosis is involved in the pathogenesis of many systemic and degenerative diseases involving multiple organ systems. It occurs when normal tissue repair, involving the activation of the coagulation cascade, inflammation, cellular proliferation, angiogenesis and tissue remodelling, becomes exaggerated and dysregulated [31]. This then evolves into a progressively irreversible fibrotic process resulting in tissue stiffening and remodelling as connective tissue replaces normal parenchymal tissue [32,33].

Epithelial cell damage, due to chronic inflammation, prolonged hypoxia or recurrent injury, results in recruitment of profibrotic cytokines and growth factors, such as TGF-β, IL-13 and platelet derived growth factor (PDGF) which in turn promote proliferation of fibroblasts [34,35]. Upon activation, fibroblasts differentiate into α-SMA expressing myofibroblasts [36]. Myofibroblasts then secrete ECM proteins, including collagens such as COL1α1 and proteoglycans such as fibronectin and vitronectin [37]. ECM is an essential component of healthy connective tissue, providing structural and biochemical support [38]. However, in excess, it results in tissue stiffness and this is compounded by myofibroblasts’ ability to contract the matrix resulting in increased tensile force and fibrosis [30,32,33,39].

### 2.2. Fibrosis in Glaucoma

Pro-fibrotic pathways, driven primarily by TGF- β are crucial in the pathophysiology of glaucoma [27]. TGF-β has been found at increased levels in the aqueous humour [40,41], optic nerve head astrocytes [42], trabecular meshwork [43] and lamina cribrosa cells of glaucomatous eyes [44]. Its role in the pro-fibrotic pathogenesis of POAG is highlighted in Table 1:

TM and LC function is heavily dependent on morphology and this is greatly affected by pro-fibrotic ECM rearrangements [61]. TGF-β induced ECM remodelling in glaucoma results in further stiffening and compression of the connective tissue microarchitecture and perpetuates the cycle of RGC damage [25,29,62]. This occurs at the LC, pinching the neurovascular supply and shearing RGC axons, and also at the TM, adding resistance to aqueous outflow, and together may further increase IOP, leading to a vicious cycle of further mechanical stretching and ECM remodelling [17,62,63,64].

Our group has shown that LC cells from glaucoma donors have characteristics of myofibroblasts [55] with increased expression of α-SMA and pro-fibrotic ECM genes and proteins (collagen 1A1, periostin, fibronectin) upon stimulation with TGF-β, cyclic stretch, and oxidative stress [44,65,66]. It also been found that thrombospondin-1, a pro-fibrotic mediator involved in cell adhesion apoptosis and ECM expression, is increased in glaucomatous LC and TM cells [65,67].

Downstream inhibitors of TGF-β have shown promising results in cell-lines and mouse models [68]. These have primarily been targeted at reducing scarring following glaucoma filtration surgery by attenuating TGF-β’s dysregulated fibroblast synthesis [69]. However, more work is required to gain consideration for use in a human population

Aging, a major risk factor for POAG development, is also a key contributor to ECM stiffness. Older tissues contain senescent fibroblasts that are resistant to apoptosis and have decreased levels of cadherin and catenin (cytoplasmic proteins that maintain epithelial integrity) [70,71]. Additionally, there is progressive collagen glycation in ageing that drives tissue stiffness and rigidity [72].

### 2.3. Mitochondrial Dysfunction

The mitochondria has many important roles in the cell. Primarily, it generates the chemical energy needed to power the cell’s biochemical reactions by producing adenosine triphosphate (ATP) through cellular pathways such as oxidative phosphorylation (OXPHOS), glycolysis and the Krebs cycle (citric acid cycle) [73,74]. It also maintains cellular homeostasis by regulating metabolic functions such as generation of reactive oxygen species (ROS), signal transduction, stress responses, maintenance of Ca^2+^ levels, proliferation and apoptosis [75,76,77]. Therefore it is no surprise that its dysfunction has been implicated in the pathogenesis of several diseases, such as cancer and neurodegenerative diseases [78,79]. Bioenergetic dysfunction may result in metabolic reprogramming, favouring the oxidation of glucose through glycolysis, even in aerobic conditions, as opposed to OXPHOS [80]. This is known as the Warburg effect. The glycolysis pathway is induced by the creation of a pseudohypoxic state and an increased AMP/ATP ratio with the demand for NAD+, a co-enzyme in redox reactions, exceeding the demand for ATP [81]. This paradoxically leads to impaired NAD+ regeneration by mitochondrial respiration and a self-reinforcing negative bioenergetic cycle. Aerobic glycolysis is a highly inefficient process producing only two ATP in addition to lactic acid, which will go on to activate TGF-β [82,83]. TGF-β1, in addition to its pro-fibrotic effects, has also been shown to directly affect mitochondrial function through ROS generation as a result of decreased complex IV activity [84].

Fibrotic tissue is highly metabolically active with evidence of fibroblastic mitochondrial dysfunction and altered cellular bioenergetics. This manifests as decreased efficiency of the electron transport chain, reduced mitochondrial macro-autophagy (mitophagy) and increased production of ROS. ROS can then drive further fibrotic change and also impair mitochondrial structure and function through aberrant mitochondrial permeability transition pore (mPTP) opening [85]. This augments a cyclical process of worsening fibrosis and bioenergetic dysfunction.

Mitochondrial dysfunction in fibrotic tissues occurs in multiple organ systems. The pro-fibrotic environment in interstitial pulmonary fibrosis (IPF) has been shown to promote mitochondrial dysfunction in pulmonary epithelial cells [86]. This manifested as decreased efficiency of electron transport chain function with increasing production of reactive oxygen species, decreased mitochondrial biogenesis and impaired mitophagy. Lactic acid levels were raised resulting in activation of TGF-β, differentiation of myofibroblasts and ultimately this drives the cycle of pulmonary fibrosis [87]. In laryngotracheal stenosis tissue, a chronic fibrotic disease, fibroblasts showed higher rates of cellular proliferation, increased glycolysis and reduced OXPHOS compared with normal fibroblasts [88,89]. Similarly, keloid fibroblast in skin scarring undergo metabolic phenotype reprogramming from oxidative phosphorylation to aerobic glycolysis (Warburg effect) with augmented glycolysis and glycolytic capacity [90]. The Warburg effect is also seen in polycystic kidney disease [91]

### 2.4. Mitochondrial Dysfunction in Glaucoma

Retinal ganglion cells, owing to their high energy requirement, are heavily dependent on mitochondria for survival and function. Evolutionary demand to optimise human vision has come at the cost of RGC bioenergetic fragility as the inner retina’s limited vascular density renders it highly vulnerable to any energy imbalances [92]. Mitochondrial dysfunction is one of the earliest detectable events within RGCs following IOP elevation in vivo in a chronic mouse model of glaucoma [93]. It has been seen in a variety of eye pathologies [94] and may be a marker of genetic predisposition in POAG [95]. The degree of bioenergetic dysfunction is associated with glaucoma susceptibility and severity and this is described at many levels in the eye and systemically [78].

#### 2.4.1. Lamina Cribrosa

Our lab recently demonstrated evidence of mitochondrial bioenergetic dysfunction in human glaucoma LC cells compared to normal controls [96]. Glaucoma LC cells were shown to have evidence of the Warburg effect, with reduced oxidative phosphorylation and ATP levels and higher aerobic glycolysis and glutaminolysis. They also displayed enhanced expression of genes associated with less efficient metabolic pathways, monocarboxylate transporters 1 and 4 (MCT1/4), glutaminase 2 (GLS2) and methylenetetra-hydrofolate dehydrogenase 2 (MTHFD2). In a separate study, our group also demonstrated elevated intracellular calcium and ROS levels, as well as increased mitochondrial number and volume density with evidence of oxidative stress in human glaucomatous LC cells [97,98,99].

#### 2.4.2. Trabecular Meshwork

Mitochondrial dysfunction has also been seen in glaucomatous TM cells [100]. This causes them to be abnormally vulnerable to Calcium (Ca^2+^) stress. Ca^2+^ overload induces mPTP opening, prompting mitochondrial release of calcium and reactive oxygen species ROS into the cytosol which, in turn, further compromises mitochondrial function and may contribute to failure of the tissue to control IOP. Ageing is itself associated with increased levels of oxidative stress in ex-vivo human TM cells [101]. This makes TM cells more prone to mitochondrial damage from insults, such as ocular hypertension, in the population at risk of POAG.

#### 2.4.3. Optic Nerve Head (ONH) + Retina

RGCs are highly reliant on mitochondria owing to their high energy requirement, with axons requiring up to 70% of all energy used by a neuron just to maintain the resting membrane potential [78,102]. It has been demonstrated that defective metabolic regulation occurs prior to evidence of neurodegeneration [78]. Mitochondrial dysfunction promotes susceptibility of RGC to a variety of stressors (raised IOP, vascular insufficiency and light exposure) [103] and has been linked with RGC loss in animal models of glaucoma [104] and in cell culture [105]. Coughlin et al. found that mitochondria in ONH in mouse were significantly smaller, with disrupted cristae and had reduced oxidative capacity [106]. Highlighting the importance of mitochondrial function in axonal survival, a study by Harun-Or-Rashid et al. showed that artificial injection of monocarboxylate transporters (MCT), a key mediator of energy balance that is under-expressed in glaucomatous RGC, improved mitochondrial function as measured by cytochrome c oxidase and succinate dehydrogenase activity [107]. Ultimately with revamped energy homeostasis, there was increased RGC density and improved electroretinogram findings (significantly greater P1 amplitude) compared to an untreated cohort. Thus, representing the huge potential for bioenergetic therapy options to combat neurodegeneration.

#### 2.4.4. Lymphoblasts/Lymphocytes (Blood Analysis)

Mitochondrial dysfunction in POAG has been described in human lymphoblasts by analysing ATP production and complex I-linked respiration, which were both markedly decreased [108]. Complex I catalyses the first step in the mitochondrial electron transport chain by oxidizing NADH transferring electrons to ubiquinone. A separate study, compared lymphoblast complex-I activity in POAG and Leber’s hereditary optic neuropathy and showed the degree of OXPHOS impairment correlated with disease severity [109]. Lascaratos et al. found that “resistant” individuals, i.e., those with high levels of IOP who did not develop glaucoma, had enhanced systemic mitochondrial efficiency and as a result were better able to withstand optic nerve injury [110]. In the lymphocytes of these individuals, they found significantly higher rates of ADP phosphorylation by mitochondrial respiratory complexes I, II and IV, hyperpolarised mitochondrial membrane potential, higher levels of mitochondrial DNA and enhanced capacity to deal with cytosolic calcium overload and exogenous oxidative stress. This indicates mitochondrial function may be a valuable disease biomarker and showcases great promise for therapeutic agents aimed at improving its function in glaucoma and also other neurodegenerative disease entities.

#### 2.4.5. Genetics

A link to mitochondrial genetic influence was first proposed when a maternal family history of POAG was found to be more likely than a paternal family history [111]. Since then, emerging research from genome wide association and other genetic studies has suggested mitochondrial DNA (mtDNA) and nuclear DNA genes that encode mitochondrial proteins contribute to the pathogenesis of POAG. Studies by Lascaratos et al. have further detailed this, linking a variety of genes (OPA1, MFN1, MFN2, CYP1B1, PARL, SOD2, SRBD1, GST, NOS3, TNFa and TP53) with mitochondrial dysfunction in POAG [112]. They reviewed the bioenergetic consequences of mtDNA and nuclear DNA derangements finding that multiple variants each contribute a small additive or multiplicative effect to the glaucomatous phenotype (‘common disease–common variant’ hypothesis). This creates a background genetic susceptibility with acquired mutations accumulating with age to negatively influence mitochondrial function. This provides a link between ageing and glaucoma in the context of bioenergetics [113].

#### 2.4.6. Bioenergetic Monitoring

Serum citrate was shown to be a potential biomarker for Glaucoma in a clinical trial at the University Hospital Basel in Switzerland [114]. Citrate is a by-product of the Krebs cycle, produced in the mitochondria and so decreased values support the idea that mitochondrial impairment is a factor in glaucoma. In addition to the use of citrate as a biomarker, mitochondrial function may be monitored directly in patient retinas using the green fluorescence emitted by oxidized flavoproteins, by-products of oxidative stress [115]. Through the monitoring of mitochondrial function, we may be able to detect the bioenergetic dysfunction that precedes apoptosis.

## 3. Metformin

Metformin is an effective, safe and inexpensive first-line pharmacotherapy for type 2 diabetes mellitus. Heralded to be an anti-aging drug [116], it also has emerged a drug of interest in combating the fibrotic changes and mitochondrial dysfunction, seen in glaucomatous eyes. It has been shown to have protective effects on ocular complications in patients with type 2 diabetes (T2DM) through its anti-inflammatory, anti-angiogenic and anti-aging effects. It reduced rates of diabetic retinopathy (DR) (25% in the metformin treatment group compared to 47% in the non-metformin group) [117], age related macular degeneration (AMD) (3.4% in the metformin group vs. 6.6% in the non-metformin group) [118] and, importantly in this discussion, glaucoma. Of note, a separate study, by Blitzer et al. on the protective role of metformin in AMD, found metformin use was associated with reduced odds of developing AMD (odds ratio (OR), 0.94 (95% CI, 0.92–0.96) in a group predominately comprised of non-diabetics (231,142 participants (74.0%)) [119]. Thus, metformin’s protective ocular mechanism appears to go beyond glycaemic control and is applicable to non-diabetics.

Lin et al. conducted a large 150, 016 patient retrospective cohort study in the United States of patients with T2DM, aged ≥40 and with no pre-existing POAG [14]. They found up to a 25% reduced risk of POAG amongst diabetics taking a high dose of metformin (>1110 g in 2 years) compared to those who took no metformin after adjusting for confounding factors (*p* = 0.02). They also noted a dose-dependent response with every 1 g increase in metformin use being associated with a 0.16% reduction in OAG risk (*p* = 0.04). Aside from its effect on glycaemic control, which they controlled for, they proposed the protective mechanisms may involve neurogenesis, inflammatory systems and longevity pathways. As age-related tissue effects changes significantly contribute to glaucoma development [4], the antiaging effect of metformin as a calorie restriction (CR) mimetic could delay the progression of disease [120]. Similar results were found in an observational study by Maleskic et al. of 234 patients with T2DM, which showed that metformin was associated with decreased odds of POAG (OR = 0.33) compared to other anti-hyperglycaemic agents (*p* = 0.017) [15]. Interestingly a separate study, in which metformin use was significantly associated with elevated IOP in participants using a high dose (>2 g/day/year), systemic use of metformin was associated with a reduced risk of POAG, in a duration-dependent manner [121]. Thus, mechanisms beyond IOP regulation are likely to be involved in the metformin-induced POAG risk-reduction. Studies which have examined metformin’s effect in ophthalmic conditions in a human population are summarized in Table 2:

No study to date has looked metformin’s effect on glaucoma in a non-diabetic group but we will examine the pathways through which it may act.

### 3.1. Metformin’s Effect on Fibrosis

#### 3.1.1. AMPK Activation

Metformin exerts its anti-fibrotic effect through a variety of signalling pathways. Its primary effect is via adenosine monophosphate protein kinase (AMPK) activation. AMPK is activated through the phosphorylation of Thr172 and this has a downstream effect of inhibition of the pro-fibrotic driver TGF-β [123]. The effects of TGF-β were discussed in Table 1. Studies have shown Metformin to prevent TGF-β associated EMT [124,125], over-expression of ECM proteins [126,127] and myofibroblast transformation [128,129].

AMPK, a critical sensor of chemical energy, may also activated by stress stimuli, such an increase in ADP:ATP ratio, ROS, hypoxia and glucose depletion [130,131] and signalling pathways including serine threonine 11 (STK11 or LKB1) and calcium/calmodulin-dependent protein kinase β (CAMMKβ) [132,133]. In addition to its anti-fibrotic effect via TGF-β inhibition, it also has downstream effects of decreased fatty acid synthesis (inhibition of acetyl-CoA carboxylase 2 (ACC2)) [134], increased autophagy (unc-51 like autophagy activating kinase 1 (ULK1) activation) [83] and glycolysis (phosphofructokinase 2 (PFK2) activation) [135]. Another key target of AMPK is the rapamycin (mTOR) signalling pathway. Whereas AMPK is active under nutrient-poor conditions and inactive under nutrient-rich conditions, mTOR is activated in the inverse pattern [136]. AMPK inhibits mTOR, through phosphorylation of Raptor resulting in decreased cellular metabolism, growth and proliferation [136]. Overall through synergistic effects, AMPK increases catabolic processes, preserving ATP, and this is key in attenuating the fibrotic process.

A laboratory study on trabecular meshwork cells in mouse models demonstrated AMPK’s role as a central regulator of ECM and cytoskeletal arrangement [137]. They examined the effect of AMPKα2 deletion and found that AMPKα2-null levels exhibited 6% higher IOP and decreased aqueous clearance compared with wild type mice (normal AMPKα2 levels), without significant differences in CCT or angle morphology. They also examined cultured human TM cells and found that activation of AMPKα led to a suppression the expression of various ECM proteins under basal and TGF-β2 stimulatory conditions. Whilst metformin is not used to drive AMPK activation in this study, the same pathway having a statistically significant effect remains promising.

Contradictory results were found by Belforte et al. who showed an association between metabolic stress in glaucoma and AMPK activation with subsequent mTORC1 inhibition [138]. They found that induced ocular hypertension in mice (*n* = 10) triggered rapid upregulation of AMPK activity and that inhibition of AMPK, with siRNA or compound C, led to RGC axonal survival (95% in treated arm vs. 77% in untreated) and restored anterograde axonal transport. Similarly, a study by Harun-Or-Rashid et al., found that AMPK was upregulated in the optic nerve and retinae of glaucomatous eyes in mouse models [139]. They hypothesized that this activation drives the NF-kB pathway which elicits a pro inflammatory mechanism. However, it remains unclear as to the significance of these results as upregulation of AMPK in response to cellular stress could be considered a beneficial, protective mechanism as it may then activate anti-fibrotic pathways.

#### 3.1.2. Other Signalling Pathways of Metformin

Independent of the AMPK pathway, Metformin has been shown to inhibit TGF-β directly [140]. It also has effects on multiple signalling pathways as detailed in Table 3:

#### 3.1.3. Metformin’s Effects on Fibrotic Tissue

Through the pathways described, metformin may be effective in a treating fibrosis in a number of organ systems. Organ systems in which metformin has been to shown to have an anti-fibrotic effect are described in Table 4:

Most recently, eye drops of metformin have been shown to prevent fibrosis after glaucoma filtration surgery in rats and human conjunctival fibroblasts by activating AMPK/Nrf2 signalling pathway [146]. Immunohistochemical staining of post-operative conjunctival tissue revealed damaged tissue showed decreased AMPK levels and upregulated α-SMA expression. They then showed treatment with metformin was as effective as 5-aminoamidazole-4-carboxamide-1-beta-D-ribofuranoside (AICAR), an established AMPK activator in laboratory studies, in decreasing TGF-β2 driven fibrosis (reduced collagen-I, fibronectin, and α-SMA expression).

### 3.2. Metformin’s Effect on Mitochondrial Function

Metformin’s effect on mitochondrial function is an area of growing research and healthy debate. Its widely regarded that metformin acts by inhibition of mitochondrial respiratory-chain complex 1 as most of metformin’s effects have been replicated by other inhibitors of Complex I [154,155,156]. The respiratory chain is a sequence of redox reactions which couple an electron flux with a vectorial transfer of protons. Complex I, a large protein complex with at least 45 subunits, includes a hydrophobic part embedded in the inner membrane involved in proton transfer and a hydrophilic part protruding into the matrix in which electrons pass from NADH to ubiquinone via a succession of redox reactions [154]. It is crucial for respiration and oxidative phosphorylation in the mammalian mitochondria

Some authors propose metformin’s inhibition is via a direct effect (having to accumulate inside the mitochondria) [157,158,159]. However, as Fontaine et al. discussed in a detailed review article, the accumulation of metformin inside the mitochondria is not supported by direct measurements, nor is it consistent with the pharmacokinetic data and would require a transporter that has not yet been discovered [154]. There is also a hypothesis that metformin triggers a signalling pathway that induces the inhibition of complex I [160]. Taken in isolation, the inhibition of complex I should decrease NADH oxidation, proton pumping across the inner mitochondrial membrane and oxygen consumption rate, resulting in lower proton gradient and reduction of proton-driven ATP synthesis from ADP [161]. However, as actual metformin concentrations in the mitochondria are in the low micromolar range, this hypothetical ATP lowering is thought not to occur with metformin therapy [162,163].

Through its inhibition of complex I, metformin also comes to act on the permeability transition pore (PTP), a channel on the inner membrane normally closed to maintain the high mitochondrial membrane potential required for ATP synthesis. In dysfunctional mitochondria, the PTP channel remains permanently opened leading to a collapse in membrane potential and a major inhibition of ATP synthesis [164]. It also inhibits complex I [165], reallocates electron flux for the production of ROS [166] and generates the release of pro-apoptotic proteins [167]. Metformin acts to inhibit this PTP opening [168] and thus has been shown to prevent cell death induced by oxidative stress in multiple cell lines [168,169]. This could be of significance in POAG, in which oxidative stress is a primary driving force. Interestingly, through the same pathway that complex I inhibition prevents PTP opening-related cell death, metformin has been shown to induce cell death in dysfunctional cells, particularly cancer cells [159,170,171]. This is explained through higher doses used and an increased sensitivity of complex I to metformin in cancer cells allowing for increased accumulation of metformin, with micromolar concentrations having a protective effect and millimolar concentrations inducing cell death.

This apparent dose dependent response of metformin’s effect on mitochondrial function may explain discrepancies in the literature between beneficial [172] and detrimental [173] effects on ATP levels, extracellular acidification and oxygen consumption rates. Wang et al. demonstrated this on hepatocyte mitochondrial function with pharmacological metformin doses (75 µM) increasing mitochondrial respiration and fission [172]. Whereas, supra-pharmacological metformin (500 and 1000 µM) inhibits mitochondrial activity by ADP reduction.

Increased mitochondrial fission, which subsequently increases mitochondrial respiratory capacity and nutrient oxidation [174], has previously been associated with prevention of ONH astrocyte dysfunction in glaucomatous neurodegeneration [102]. In multiple studies at lower levels, closer to a clinically relevant dose, metformin has been shown to activate mitochondrial respiratory chain activity [175,176].

Vastly different dynamics of response to metformin exist in vivo and in tissue culture owing to variable conditions and metabolic rates. One particular analysis of metformin levels in colorectal cancer cells treated in vitro showed intracellular accumulation levels of only 10%–15% of the incubated dose of the drug [177]. PET studies of 11C-labelled metformin have also shown that that plasma bioavailability remains well below the concentrations seen in vitro studies on proliferation effects [178]. Even at a high dose of 1000 µM, enzymatic activity of mitochondrial complexes I–V has been shown to be unaffected as metformin concentration in the mitochondria (64.5 µM) falls far below the IC_50_ (19–66 mM) for mitochondrial complex I [172]. This indicates supra-therapeutic metformin’s seemingly harmful effect is not via direct action, as previously discussed, but may be through reduction of ADP with the mitochondrial membrane potential being unable to generate ATP as a result. This could theoretically even have a beneficial effect in that reduced mitochondrial OXPHOS would ultimately result in AMPK-mediated activation of catabolic pathways and inhibition of anabolic processes through its regulation of mTORC1. This highlights the paradox of metformin’s inhibition of Complex-I and the importance of pharmacological concentrations used.

### 3.3. NAD/NADH

The biphasic nature of metformin extends to its effect on NAD/NADH. Alshawi et al. found that a low dose of metformin (therapeutic equivalent: <2 nmol/mg) caused a more oxidized mitochondrial NADH/NAD state (3-hydroxybutyrate/acetoacetate ratio) and an increase in lactate/pyruvate ratio (more reduced cytoplasmic NADH/NAD), whereas a higher metformin dose (>5 nmol/mg) caused a more reduced mitochondrial NADH/NAD state, similar to complex I inhibition by rotenone [179]. As complex I is responsible for oxidizing NADH in the mitochondrial matrix, this is another example of metformin’s dose dependent effect on the inhibition of complex I. Metformin is unique in this regarded amongst anti-hyperglycaemic guanides as Cameron et al. showed [180]. They found that, whilst other guanide derivatives (DG5-DG10 and Phenformin) caused the NADH/NAD+ couple to become more reduced resulting in mitochondrial deterioration over time, metformin exerted a selective oxidation of the mitochondrial NADH/NAD+ couple.

This NADH oxidation, seen with low levels of metformin, produces two electrons that reduce ubiquinone to ubiquinol in the mitochondrial inner membrane, supplying respiratory complexes III and IV with electrons for the reduction of O2 to water [158]. Subsequently this energy derived from the NADH:ubiquinone redox reaction is used to transport protons across the inner membrane, supporting the proton-motive force that drives ATP synthesis by F_1_F_O_-ATP synthase.

NAD+ is an essential coenzyme in four steps of the Krebs cycle, in the glyceraldehyde 3-phosphate dehydrogenase (GAPDH) step of glycolysis, in the conversion of lactate into pyruvate and, of importance in POAG, acts as a redox cofactor and metabolite essential for neuronal survival [181]. In addition to raised levels being seen with metformin treatment, NAD+ may be naturally regenerated by nicotinamide mononucleotide adenylyltransferase-3 (NMNAT3) as a protective mechanism against the mitochondrial dysfunction that precedes axon degeneration in glaucoma [182,183]. It may also be supplemented through the use of vitamin B3, nicotinamide, a precursor in NAD+ synthesis [184]. Repleting NAD, through vitamin B3 or metformin, improves mitochondrial function allowing for recovery of retinal ganglion cell function and ultimately protection of visual function in POAG and AMD [181,185]. The Pete Williams lab has done extensive research into bioenergetic insufficiency driving neurodegeneration and novel therapeutic approaches to address this. They have examined NAD’s effect in rodent models with induced, isolated ocular hypertensive, axon degenerative, and mitochondrial degenerative insults [186]. The use of nicotinamide supplementation in this study provided neuroprotective effects by increasing oxidative phosphorylation and mitochondrial size and motility, as well as by buffering and preventing metabolic stress [186].

## 4. Future and Conclusions

One-third of POAG patients have normal IOP, whereas one-third of patients with high IOP do not develop glaucoma [187,188]. Understanding this makes it abundantly clear that there is a need to evolve our arsenal in glaucoma management beyond simply lowering IOP. Glaucoma is a multifactorial disease entity that must be addressed as such. Targeting the pro-fibrotic environment and mitochondrial dysfunction in glaucoma represents a very promising avenue for pharmacological advances in glaucoma management.

Metformin, a widely available and cheap drug, targets both mechanisms and has shown very favourable results in experimental models and observational studies. However, the exact molecular mechanism of its action remains partly unknown despite its use for over 60 years. The continued exploration AMPK’s role as a key metabolic sensor, particularly in LC and TM cells in glaucoma, will help us further understand the driving force underlying fibrotic changes in the eye. Additionally, the exact nature of the mitochondrial interaction between the drug and complex 1 remains to be elucidated. More research is also needed to ascertain metformin’s effect on NAD+ recycling. Further knowledge of its precise mechanism of action in POAG are necessary to untangle the link between its cause and effect.

Furthermore, there remains the question of drug delivery. Whilst favourable results have been shown in cross-sectional studies involving oral metformin use, it is not a medication bereft of side-effects. Targeted therapies should always be explored to minimize these unwanted systemic side effects. Intravitreal injections of metformin have been successfully trialled in mouse models where it was shown to significantly reduce apoptosis in photoreceptors [189]. This was achieved through its liquification in phosphate buffer and represents a promising avenue of future research with the goal of application to a human population. Topical eye drop formulations of metformin have also been successfully trialled in rat models where it was shown to prevent fibrosis following glaucoma filtration surgery [146]. Finally, we must also gain a firm understanding of what metformin exposure in cellular models is equivalent of therapeutic exposure in a human population. However, with a clinical trial [190] already underway on its effect on visual function in human patients with glaucoma, the metformin story will be worth following.

## Figures and Tables

**Table 1:** Role of TGF-β in POAG.

Effect	Mechanism	Reference
↑ Fibroblasts	TGF-β1-mediated chemotaxis has been shown to promote fibroblast proliferation and recruitment. In rat models, this has been shown to induce anterior segment changes and subsequently raise IOP	[45,46]
↑ ECM production	Facilitates excessive ECM deposition in the TM and LC through over-production of fibronectin and collagen I and IV in patients with POAG	[47,48,49]
↓ ECM degradation	Upregulates plasminogen activator inhibitor-1 (PAI-1) which has been found to have increased expression in the aqueous humour of human glaucomatous eyes. Additionally it elicits induction of tissue inhibitors of matrix metalloproteinases (TIMPs) in optic nerve head astrocytes in POAG. Both act to inhibit ECM degradation.	[50,51]
Epithelial-mesenchymal transition	Mediates epithelial-mesenchymal transition (EMT) whereby epithelial cells adopt a mesenchymal phenotype with elevated resistance to apoptosis and greatly increased production of ECM components. This results in aberrant aqueous humour outflow in POAG	[52,53,54]
Myofibroblast transformation	Transforms fibroblasts into contractile myofibroblasts through downstream signalling, activating canonical WNT signalling and decreasing peroxisome proliferator-activated receptor (PPARγ). A myofibroblastic phenotype has been shown in the LC of glaucomatous eyes and this contributes to disrupted axoplasmic flow	[52,55]
ECM cross-linking	Induces expression of tissue transglutaminase (tTgase) which irreversibly cross-links ECM proteins and enhances tissue stiffness in the TM. This further increases outflow resistance in POAG	[56]
Cell-Cell Adhesion	Enhances N-cadherin and β-catenin mediated cell-cell adhesion in human glaucomatous TM cells, further adding to its biomechanical alteration	[57,58]
Cyclical process	TGF-β release is stimulated by ECM rigidity and therefore the fibrotic process becomes a self-reinforcing cycle in POAG	[59,60]

**Table 2:** Studies of metformin’s effect on a variety of ocular conditions.

Study Title	Study Design + Results	Reference
Association of Geroprotective Effects of Metformin and Risk of Open-Angle Glaucoma in Persons with Diabetes Mellitus	Retrospective cohort study of 150,016 patients with T2DM and no record of POAG ⇒ 25% reduced risk of POAG in those taking a high dose of metformin (>1110 g in 2 years) compared to those who took no metformin (*p* = 0.02) ⇒ Every 1 g increase in metformin use was associated with a 0.16% reduction in POAG risk (*p* = 0.04)	[14]
Metformin Use Associated with Protective Effects for Ocular Complications in Patients with Type 2 Diabetes—Observational Study	Observational study of 234 patients with T2DM (190 using metformin and 44 using other oral anti-hyperglycaemics) ⇒ Metformin use was associated with decreased odds of POAG (*p* = 0.006) and DR (*p* = 0.017) development compared to other anti-hyperglycaemic agents	[15]
Association of Metformin Treatment with Reduced Severity of Diabetic Retinopathy in Type 2 Diabetic Patients	Retrospective chart review of 335 patients with DR ⇒ Long term use of metformin was independently associated with lower rates of severe non-proliferative DR or proliferative DR (*p* < 0.001)	[117]
Association Between Metformin and a Lower Risk of Age-Related Macular Degeneration in Patients with Type 2 Diabetes	Retrospective cohort study of 68,205 subjects (45,524 taking metformin and 22,681 non-users) ⇒ Metformin group had a significantly lower risk of AMD (*p* < 0.05) ⇒ Lower hazard ratios with increasing total and average doses (*p* < 0.05)	[118]
The Common Antidiabetic Drug Metformin Reduces Odds of Developing Age-Related Macular Degeneration	Retrospective case-control study of 7788 age matched patients (1947 with AMD and 5841 without AMD) ⇒ Metformin use was associated with decreased odds of developing AMD (*p* < 0.001) ⇒ Other medications (anti-hyperglycaemics and statins) were not associated with decreased odds of developing AMD	[122]
Association of Metformin Use With Age-Related Macular Degeneration: A Case-Control Study	Retrospective case-control study of 624,780 patients (312,404 with AMD and 312,376 controls) ⇒ Metformin use was associated with reduced odds of developing AMD (*p* < 0.01) in a dose-dependent manner ⇒ This was shown in a cohort of predominantly non-diabetics (74.0%)	[119]

**Table 3:** Anti-fibrotic signalling pathways of metformin (independent of AMPK).

Pathway	Mechanism	Reference
PPAR-γ and BMP2 (bone morphogenetic protein 2) activation	Induces lipogenic differentiation resulting in the transformation of myofibroblasts into lipofibroblasts	[128]
mTORC inhibition	Leads to an anti-tumorigenesis effect—↓ cell proliferation, angiogenesis, actin remodelling and glucose uptake	[116,141,142]
Smad 2/3 inhibition	Impedes excessive ECM synthesis via connective tissue growth factor (CTGF)—↓ COL1α1 and α-SMA ⇒ *Downstream mediator of TGF-β1 signalling (canonical)* ⇒ *Inhibition* via *AMPK activation*	[125,126,127,140,143]
ERK 1/2 (extracellular signal-regulated protein kinase 1/2) inhibition	Impedes excessive ECM synthesis—↓ COL1α1 and α-SMA ⇒ *Downstream mediator of TGF-β1 signalling (non-canonical)* ⇒ *Inhibition* via *AMPK activation*	[126,143]
NOX-4 (NADPH Oxidase 4) suppression	Prevents production of ROS (mediator of TGF-β)—critical for Smad phosphorylation and myofibroblast differentiation ⇒ *Inhibition* via *AMPK activation*	[144]
Nrf2 (nuclear factor erythroid 2-related factor 2) activation	Results in kelch-like ECH-associated protein 1 (KEAP1) degradation and associated ↑ level of transcription factors related to anti-inflammatory, anti-oxidant and other cell protection pathways	[145,146,147]

**Table 4:** Anti-fibrotic effects of metformin in multiple organ systems.

Organ System	Mechanism	Reference
PulmonaryFibrosis	*Pathway:* Inhibition of the TGF-β1 induced activation of Smad 2/3, signal transducer and activator of transcription 3 (STAT3), protein kinase B (AKT) and ERK 1/2 signalling pathway *Results:* ⇒ ↓ COL1α1 and α-actin	[126]
	*Pathway:* Activation of PPARγ and BMP2 Inhibition of TGF-β1 *Results:* ⇒ Induces lipogenic differentiation in myofibroblasts ⇒ ↓ COL1α1	[128]
	*Pathway:* Activation of AMPK *Results:* ⇒ ↓ COL1α1 and α-SMA ⇒ ↑ myofibroblast sensitivity to intrinsic apoptosis ⇒ Enhanced mitochondrial biogenesis: ↑ NADH ubiquinone dehydrogenase 1β subcomplex 8 (NDFUB8) and mitochondrial transcription factor-A	[148]
RenalFibrosis	*Pathway:* Activation of AMPK *Results:* ⇒ ↓ α-SMA and fibronectin ⇒ Prevented increased tumour necrosis factor α (TNFα) and vascular cell adhesion molecule 1 (VCAM1) expression (inflammation) ⇒ ↓ TGF-β1	[149]
	*Pathway:* Activation of AMPK *Results:* ⇒ ↓ Endoplasmic Reticulum stress and EMT	[150]
	*Pathway:* Activation of deptor (an endogenous negative regulator of mTOR) *Results:* ⇒ ↓ CD68, αSMA, p-MTOR and p-p70S6K	[151]
Cardiac Fibrosis	*Pathway:* Activation of AMPK *Results:* ⇒ Inhibition of aldosterone-induced fibroblast activation, proliferation and migration ⇒ ↓TRAF3IP2 (ROS) and ↓ IL-6, 17, 18 (Inflammation)	[152]
	*Pathway:* Inhibition of the TGF-β1-Smad 3 signalling pathway *Results:* ⇒ ↓ TGF-β1 ⇒ ↓ COL1α1 ⇒ Inhibition of the nuclear translocation and transcriptional activity of Smad3	[153]
Peritendinous Fibrosis	*Pathway:* Activation of AMPK Inhibition of TGF-β1, Smad 2/3 and ERK 1/2 signalling *Effect:* ⇒ ↓ COL1α1, COL3α1 and α-SMA	[143]

## References

[1] Weinreb R.N., Aung T., Medeiros F.A. (2014). The Pathophysiology and Treatment of Glaucoma. JAMA.

[2] Quigley H.A. (2006). The number of people with glaucoma worldwide in 2010 and 2020. Br. J. Ophthalmol..

[3] Tham Y.-C., Li X., Wong T.Y., Quigley H.A., Aung T., Cheng C.-Y. (2014). Global Prevalence of Glaucoma and Projections of Glaucoma Burden through 2040. Ophthalmology.

[4] Guedes G., Tsai J.C., Loewen N. (2011). Glaucoma and Aging. Curr. Aging Sci..

[5] Coleman A.L., Miglior S. (2008). Risk Factors for Glaucoma Onset and Progression. Surv. Ophthalmol..

[6] Le A., Mukesh B.N., McCarty C., Taylor H. (2003). Risk Factors Associated with the Incidence of Open-Angle Glaucoma: The Visual Impairment Project. Investig. Ophthalmol. Vis. Sci..

[7] Crawley L., Zamir S.M., Cordeiro M.F., Guo L. (2012). Clinical Options for the Reduction of Elevated Intraocular Pressure. Ophthalmol. Eye Dis..

[8] Heijl A., Leske M.C., Bengtsson B., Hyman L., Hussein M. (2002). Reduction of Intraocular Pressure and Glaucoma Progression. Arch. Ophthalmol..

[9] Leske M.C., Heijl A., Hussein M., Bengtsson B., Hyman L., Komaroff E. (2003). Factors for Glaucoma Progression and the Effect of Treatment. Arch. Ophthalmol..

[10] Newman-Casey P.A., Robin A.L., Blachley T., Farris K., Heisler M., Resnicow K., Lee P.P. (2015). The Most Common Barriers to Glaucoma Medication Adherence. Ophthalmology.

[11] McKinnon S.J., Goldberg L.D., Peeples P., Walt J.G., Bramley T.J. (2008). Current management of glaucoma and the need for complete therapy. Am. J. Manag. Care.

[12] Cho H.-K., Kee C. (2014). Population-based glaucoma prevalence studies in Asians. Surv. Ophthalmol..

[13] Collaborative Normal-Tension Glaucoma Study Group (1998). The effectiveness of intraocular pressure reduction in the treatment of normal-tension glaucoma. Am. J. Ophthalmol..

[14] Lin H.-C., Stein J., Nan B., Childers D., Newman-Casey P.A., Thompson D., Richards J.E. (2015). Association of Geroprotective Effects of Metformin and Risk of Open-Angle Glaucoma in Persons With Diabetes Mellitus. JAMA Ophthalmol..

[15] Maleškić S., Kusturica J., Gušić E., Rakanović-Todić M., Šečić D., Burnazović-Ristić L., Kulo A. (2018). Metformin use associated with protective effects for ocular complications in patients with type 2 diabetes – observational study. Acta Med. Acad..

[16] Guo L., Moss S.E., Alexander R.A., Ali R., Fitzke F.W., Cordeiro M.F. (2005). Retinal Ganglion Cell Apoptosis in Glaucoma Is Related to Intraocular Pressure and IOP-Induced Effects on Extracellular Matrix. Investig. Ophthalmol. Vis. Sci..

[17] Vranka J.A., Kelley M.J., Acott T.S., Keller K.E. (2015). Extracellular matrix in the trabecular meshwork: Intraocular pressure regulation and dysregulation in glaucoma. Exp. Eye Res..

[18] Buffault J., Labbé A., Hamard P., Brignole-Baudouin F., Baudouin C. (2020). The trabecular meshwork: Structure, function and clinical implications. A review of the literature. J. Fr. Ophtalmol..

[19] Quigley H.A., Addicks E.M. (1981). Regional Differences in the Structure of the Lamina Cribrosa and Their Relation to Glaucomatous Optic Nerve Damage. Arch. Ophthalmol..

[20] Mead B., Tomarev S. (2016). Evaluating retinal ganglion cell loss and dysfunction. Exp. Eye Res..

[21] Quigley H.A., Addicks E.M., Green W.R., Maumenee A.E. (1981). Optic Nerve Damage in Human Glaucoma. Arch. Ophthalmol..

[22] Fechtner R.D., Weinreb R.N. (1994). Mechanisms of optic nerve damage in primary open angle glaucoma. Surv. Ophthalmol..

[23] Sigal I.A., Flanagan J.G., Tertinegg I., Ethier C.R. (2007). Predicted extension, compression and shearing of optic nerve head tissues. Exp. Eye Res..

[24] Yan D.B., Coloma F.M., Metheetrairut A., Trope G.E., Heathcote J.G., Ethier C.R. (1994). Deformation of the lamina cribrosa by elevated intraocular pressure. Br. J. Ophthalmol..

[25] Quigley H.A., Hohman R.M., Addicks E.M., Massof R.W., Green W.R. (1983). Morphologic Changes in the Lamina Cribrosa Correlated with Neural Loss in Open-Angle Glaucoma. Am. J. Ophthalmol..

[26] Hernandez M.R., Andrzejewska W.M., Neufeld A.H. (1990). Changes in the Extracellular Matrix of the Human Optic Nerve Head in Primary Open-Angle Glaucoma. Am. J. Ophthalmol..

[27] Zhavoronkov A., Izumchenko E., Kanherkar R.R., Teka M., Cantor C., Manaye K., Sidransky D., West M.D., Makarev E., Csoka A.B. (2016). Pro-fibrotic pathway activation in trabecular meshwork and lamina cribrosa is the main driving force of glaucoma. Cell Cycle.

[28] McDonnell F., O’Brien C., Wallace D. (2014). The Role of Epigenetics in the Fibrotic Processes Associated with Glaucoma. J. Ophthalmol..

[29] Hopkins A., Murphy R., Irnaten M., Wallace D.M., Quill B., O’Brien C. (2020). The role of lamina cribrosa tissue stiffness and fibrosis as fundamental biomechanical drivers of pathological glaucoma cupping. Am. J. Physiol. Physiol..

[30] Wynn T.A., Ramalingam T.R. (2012). Mechanisms of fibrosis: Therapeutic translation for fibrotic disease. Nat. Med..

[31] White E.S., Mantovani A. (2012). Inflammation, wound repair, and fibrosis: Reassessing the spectrum of tissue injury and resolution. J. Pathol..

[32] Wynn T.A. (2007). Cellular and molecular mechanisms of fibrosis. J. Pathol..

[33] Tomasek J.J., Gabbiani G., Hinz B., Chaponnier C., Brown R.A. (2002). Myofibroblasts and mechano-regulation of connective tissue remodelling. Nat. Rev. Mol. Cell Biol..

[34] Li M.O., Wan Y.Y., Sanjabi S., Robertson A.-K.L., Flavell R.A. (2006). Transforming growth factor-β regulation of immune responses. Annu. Rev. Immunol..

[35] Wynn T.A. (2003). IL-13 effectorfunctions. Annu. Rev. Immunol..

[36] Gabbiani G. (2003). The myofibroblast in wound healing and fibrocontractive diseases. J. Pathol..

[37] Klingberg F., Hinz B., White E. (2012). The myofibroblast matrix: Implications for tissue repair and fibrosis. J. Pathol..

[38] Papageorgis P. (2015). TGFβSignaling in Tumor Initiation, Epithelial-to-Mesenchymal Transition, and Metastasis. J. Oncol..

[39] Hinz B., Phan S., Thannickal V.J., Galli A., Bochaton-Piallat M.-L., Gabbiani G. (2007). The Myofibroblast: One Function, Multiple Origins. Am. J. Pathol..

[40] Agarwal P., Daher A.M., Agarwal R. (2015). Aqueous humor TGF-beta2 levels in patients with open-angle glaucoma: A meta-analysis. Mol. Vis..

[41] Tripathi R.C., Li J., Chan W.A., Tripathi B.J. (1994). Aqueous Humor in Glaucomatous Eyes Contains an Increased Level of TGF-β2. Exp. Eye Res..

[42] Zode G.S., Sethi A., Brun-Zinkernagel A.M., Chang I.F., Clark A.F., Wordinger R.J. (2011). Transforming growth factor-beta2 increases extracellular matrix proteins in optic nerve head cells via activation of the Smad signaling pathway. Mol. Vis..

[43] Fleenor D.L., Shepard A.R., Hellberg P.E., Jacobson N., Pang I.-H., Clark A.F. (2006). TGFβ2-Induced Changes in Human Trabecular Meshwork: Implications for Intraocular Pressure. Investig. Ophtalmol. Vis. Sci..

[44] Kirwan R.P., Leonard M.O., Murphy M., Clark A.F., O’Brien C.J. (2005). Transforming growth factor-β-regulated gene transcription and protein expression in human GFAP-negative lamina cribrosa cells. Glia.

[45] Epstein F.H., Border W.A., Noble N.A. (1994). Transforming Growth Factor β in Tissue Fibrosis. N. Engl. J. Med..

[46] Robertson J.V., Golesic E., Gauldie J., West-Mays J.A. (2010). Ocular Gene Transfer of Active TGF-β Induces Changes in Anterior Segment Morphology and Elevated IOP in Rats. Investig. Ophtalmol. Vis. Sci..

[47] Medina-Ortiz W.E., Belmares R., Neubauer S., Wordinger R., Clark A.F. (2013). Cellular Fibronectin Expression in Human Trabecular Meshwork and Induction by Transforming Growth Factor-β2. Investig. Ophtalmol. Vis. Sci..

[48] Faralli J.A., Filla M.S., Peters D.M. (2019). Role of Fibronectin in Primary Open Angle Glaucoma. Cells.

[49] Fukuchi T., Ueda J., Abe H., Sawaguchi S. (2001). Cell adhesion glycoproteins in the human lamina cribrosa. Jpn. J. Ophthalmol..

[50] Dan J., Belyea D., Gertner G., Leshem I., Lusky M., Miskin R. (2005). Plasminogen Activator Inhibitor-1 in the Aqueous Humor of Patients With and Without Glaucoma. Arch. Ophthalmol..

[51] Agapova O.A., Ricard C.S., Salvador-Silva M., Hernandez M.R. (2001). Expression of matrix metalloproteinases and tissue inhibitors of metalloproteinases in human optic nerve head astrocytes. Glia.

[52] Vallée A., LeCarpentier Y. (2019). TGF-β in fibrosis by acting as a conductor for contractile properties of myofibroblasts. Cell Biosci..

[53] Kalluri R., Neilson E.G. (2003). Epithelial-mesenchymal transition and its implications for fibrosis. J. Clin. Investig..

[54] Takahashi E., Inoue T., Fujimoto T., Kojima S., Tanihara H. (2014). Epithelial mesenchymal transition-like phenomenon in trabecular meshwork cells. Exp. Eye Res..

[55] Liu B., Kilpatrick J.I., Lukasz B., Jarvis S.P., McDonnell F., Wallace D., Clark A.F., O’Brien C.J. (2018). Increased Substrate Stiffness Elicits a Myofibroblastic Phenotype in Human Lamina Cribrosa Cells. Investig. Ophthalmol. Vis. Sci..

[56] Welge-Lüssen U., May C.A., Lütjen-Drecoll E. (2000). Induction of tissue transglutaminase in the trabecular meshwork by TGF-beta1 and TGF-beta2. Investig. Ophthalmol. Vis. Sci..

[57] Wecker T., Han H., Grehn F., Schlunck G., Börner J. (2013). Effects of TGF- 2 on Cadherins and -Catenin in Human Trabecular Meshwork Cells. Investig. Ophthalmol. Vis. Sci..

[58] Webber H.C., Bermudez J.Y., Millar J.C., Mao W., Clark A.F. (2018). The Role of Wnt/β-Catenin Signaling and K-Cadherin in the Regulation of Intraocular Pressure. Investig. Ophthalmol. Vis. Sci..

[59] Froese A.R., Shimbori C., Bellaye P.-S., Inman M., Obex S., Fatima S., Jenkins G., Gauldie J., Ask K., Kolb M. (2016). Stretch-induced Activation of Transforming Growth Factor-β1in Pulmonary Fibrosis. Am. J. Respir. Crit. Care Med..

[60] Fuchshofer R., Tamm E.R. (2012). The role of TGF-β in the pathogenesis of primary open-angle glaucoma. Cell Tissue Res..

[61] Sander E.A., Downs J.C., Hart R.T., Burgoyne C.F., Nauman E.A. (2006). A Cellular Solid Model of the Lamina Cribrosa: Mechanical Dependence on Morphology. J. Biomech. Eng..

[62] Hernandez M.R., Ye H. (1993). Glaucoma: Changes in Extracellular Matrix in the Optic Nerve Head. Ann. Med..

[63] Braunger B.M., Fuchshofer R., Tamm E.R. (2015). The aqueous humor outflow pathways in glaucoma: A unifying concept of disease mechanisms and causative treatment. Eur. J. Pharm. Biopharm..

[64] Wallace D.M., Murphy-Ullrich J.E., Downs J.C., O’Brien C.J. (2014). The role of matricellular proteins in glaucoma. Matrix Biol..

[65] Kirwan R.P., Wordinger R., Clark A.F., O’Brien C.J. (2009). Differential global and extra-cellular matrix focused gene expression patterns between normal and glaucomatous human lamina cribrosa cells. Mol. Vis..

[66] Kirwan R.P., Felice L., Clark A.F., Leonard M.O., O’Brien C.J. (2012). Hypoxia Regulated Gene Transcription in Human Optic Nerve Lamina Cribrosa Cells in Culture. Investig. Ophthalmol. Vis. Sci..

[67] Flügel-Koch C., Ohlmann A., Fuchshofer R., Welge-Lüssen U., Tamm E.R. (2004). Thrombospondin-1 in the trabecular meshwork: Localization in normal and glaucomatous eyes, and induction by TGF-β1 and dexamethasone in vitro. Exp. Eye Res..

[68] Prendes M.A., Harris A., Wirostko B.M., Gerber A.L., Siesky B. (2013). The role of transforming growth factor β in glaucoma and the therapeutic implications. Br. J. Ophthalmol..

[69] Meyer-Ter-Vehn T., Gebhardt S., Sebald W., Buttmann M., Grehn F., Schlunck G., Knaus P. (2006). p38 Inhibitors Prevent TGF-β–Induced Myofibroblast Transdifferentiation in Human Tenon Fibroblasts. Investig. Ophthalmol. Vis. Sci..

[70] Hampel B., Wagner M., Teis D., Zwerschke W., Huber L.A., Jansen-Durr P. (2005). Apoptosis resistance of senescent human fibroblasts is correlated with the absence of nuclear IGFBP-3. Aging Cell.

[71] Parrish A.R. (2017). The impact of aging on epithelial barriers. Tissue Barriers.

[72] Snedeker J.G., Gautieri A. (2014). The role of collagen crosslinks in ageing and diabetes—The good, the bad, and the ugly. Muscles Ligaments Tendons J..

[73] Osellame L.D., Blacker T.S., Duchen M.R. (2012). Cellular and molecular mechanisms of mitochondrial function. Best Pr. Res. Clin. Endocrinol. Metab..

[74] Lee J.-H.Y.M. (2015). Metabolic interplay between glycolysis and mitochondrial oxidation: The reverse Warburg effect and its therapeutic implication. World J. Biol. Chem..

[75] Soubannier V., McBride H.M. (2009). Positioning mitochondrial plasticity within cellular signaling cascades. Biochim. Biophys. Acta (BBA) Bioenerg..

[76] Hausenloy D.J., Ruiz-Meana M. (2010). Not just the powerhouse of the cell: Emerging roles for mitochondria in the heart. Cardiovasc. Res..

[77] Nunnari J., Suomalainen A. (2012). Mitochondria: In Sickness and in Health. Cell.

[78] Inman D.M., Harun-Or-Rashid M. (2017). Metabolic Vulnerability in the Neurodegenerative Disease Glaucoma. Front. Neurosci..

[79] Pavlides S., Vera I., Gandara R., Sneddon S., Pestell R.G., Mercier I., Martinez-Outschoorn U., Whitaker-Menezes D., Howell A., Sotgia F. (2012). Warburg Meets Autophagy: Cancer-Associated Fibroblasts Accelerate Tumor Growth and Metastasis via Oxidative Stress, Mitophagy, and Aerobic Glycolysis. Antioxid. Redox Signal..

[80] Warburg O., Wind F., Negelein E. (1927). The metabolism of tumors in the body. J. Gen. Physiol..

[81] Luengo A., Li Z., Gui D.Y., Sullivan L.B., Zagorulya M., Do B.T., Ferreira R., Naamati A., Ali A., Lewis C.A. (2020). Increased demand for NAD+ relative to ATP drives aerobic glycolysis. Mol. Cell.

[82] Martinez-Outschoorn U.E., Lin Z., Trimmer C., Flomenberg N., Wang C., Pavlides S., Pestell R.G., Howell A., Sotgia F., Lisanti M.P. (2011). Cancer cells metabolically “fertilize” the tumor microenvironment with hydrogen peroxide, driving the Warburg effect. Cell Cycle.

[83] Kim J., Kundu M., Viollet B., Guan K.-L. (2011). AMPK and mTOR regulate autophagy through direct phosphorylation of Ulk1. Nature.

[84] Yoon Y.-S., Lee J.-H., Hwang S.C., Choi K.S., Yoon G. (2005). TGF β1 induces prolonged mitochondrial ROS generation through decreased complex IV activity with senescent arrest in Mv1Lu cells. Oncogene.

[85] Li X., Zhang W., Cao Q., Wang Z., Zhao M., Xu L., Zhuang Q. (2020). Mitochondrial dysfunction in fibrotic diseases. Cell Death Discov..

[86] Zank D.C., Bueno M., Mora A.L., Rojas M. (2018). Idiopathic Pulmonary Fibrosis: Aging, Mitochondrial Dysfunction, and Cellular Bioenergetics. Front. Med..

[87] Kottmann R.M., Kulkarni A.A., Smolnycki K.A., Lyda E., Dahanayake T., Salibi R., Honnons S., Jones C., Isern N., Hu J.Z. (2012). Lactic Acid Is Elevated in Idiopathic Pulmonary Fibrosis and Induces Myofibroblast Differentiation via pH-Dependent Activation of Transforming Growth Factor-β. Am. J. Respir. Crit. Care Med..

[88] Ma G., Samad I., Motz K., Yin L., Ba M.V.D., Ding D., Bs D.R.N., Elisseeff J.H., Horton M.R., Hillel A.T. (2016). Metabolic variations in normal and fibrotic human laryngotracheal-derived fibroblasts: A Warburg-like effect. Laryngoscope.

[89] Murphy M.K., Motz K.M., Ding D., Yin L., Duvvuri M., Bs M.F., Hillel A.T. (2017). Targeting metabolic abnormalities to reverse fibrosis in iatrogenic laryngotracheal stenosis. Laryngoscope.

[90] Li Q., Qin Z., Nie F., Bi H., Zhao R., Pan B., Ma J., Xie X. (2018). Metabolic reprogramming in keloid fibroblasts: Aerobic glycolysis and a novel therapeutic strategy. Biochem. Biophys. Res. Commun..

[91] Riwanto M., Kapoor S., Rodriguez D., Edenhofer I., Segerer S., Wüthrich R.P. (2016). Inhibition of Aerobic Glycolysis Attenuates Disease Progression in Polycystic Kidney Disease. PLoS ONE.

[92] Casson R.J., Chidlow G., Crowston J.G., Williams P.A., Wood J.P. (2020). Retinal energy metabolism in health and glaucoma. Prog. Retin. Eye Res..

[93] Williams P.A., Harder J.M., John S.W. (2017). Glaucoma as a Metabolic Optic Neuropathy. J. Glaucoma.

[94] Kamel K., Farrell M., O’Brien C. (2017). Mitochondrial dysfunction in ocular disease: Focus on glaucoma. Mitochondrion.

[95] Khawaja A., Bailey J.C., Kang J.H., Allingham R.R., Hauser M.A., Brilliant M., Budenz D.L., Christen W.G., Fingert J., Gaasterland D. (2016). Assessing the Association of Mitochondrial Genetic Variation With Primary Open-Angle Glaucoma Using Gene-Set Analyses. Investig. Ophtalmol. Vis. Sci..

[96] Kamel K., O’Brien C.J., Zhdanov A.V., Papkovsky D.B., Clark A.F., Stamer W.D., Irnaten M. (2020). Reduced Oxidative Phosphorylation and Increased Glycolysis in Human Glaucoma Lamina Cribrosa Cells. Investig. Ophtalmol. Vis. Sci..

[97] McElnea E., Quill B., Docherty N., Irnaten M., Siah W., Clark A., O’Brien C., Wallace D. (2011). Oxidative stress, mitochondrial dysfunction and calcium overload in human lamina cribrosa cells from glaucoma donors. Mol. Vis..

[98] Irnaten M., Zhdanov A., Brennan D., Crotty T., Clark A., Papkovsky D., O’Brien C. (2018). Activation of the NFAT–Calcium Signaling Pathway in Human Lamina Cribrosa Cells in Glaucoma. Investig. Ophthalmol. Vis. Sci..

[99] McElnea E.M., Hughes E., McGoldrick A., McCann A., Quill B., Docherty N.G., Irnaten M., Farrell M., Clark A.F., O’Brien C.J. (2014). Lipofuscin accumulation and autophagy in glaucomatous human lamina cribrosa cells. BMC Ophthalmol..

[100] He Y., Ge J., Tombran-Tink J. (2008). Mitochondrial Defects and Dysfunction in Calcium Regulation in Glaucomatous Trabecular Meshwork Cells. Investig. Ophthalmol. Vis. Sci..

[101] Pulliero A., Seydel A., Camoirano A., Saccà S.C., Sandri M., Izzotti A. (2014). Oxidative Damage and Autophagy in the Human Trabecular Meshwork as Related with Ageing. PLoS ONE.

[102] Ju W., Kim K., Noh Y.H., Hoshijima M., Lukas T.J., Ellisman M.H., Weinreb R.N., Perkins G.A. (2014). Increased mitochondrial fission and volume density by blocking glutamate excitotoxicity protect glaucomatous optic nerve head astrocytes. Glia.

[103] Kong G.Y.X., Van Bergen N.J., Trounce I., Crowston J.G. (2009). Mitochondrial Dysfunction and Glaucoma. J. Glaucoma.

[104] Ju W.-K., Kim K.-Y., Angert M., Duong-Polk K.X., Lindsey J.D., Ellisman M.H., Weinreb R.N. (2009). Memantine Blocks Mitochondrial OPA1 and CytochromecRelease and Subsequent Apoptotic Cell Death in Glaucomatous Retina. Investig. Ophthalmol. Vis. Sci..

[105] Ju W.-K., Liu Q., Kim K.-Y., Crowston J.G., Lindsey J.D., Agarwal N., Ellisman M.H., Perkins G.A., Weinreb R.N. (2007). Elevated Hydrostatic Pressure Triggers Mitochondrial Fission and Decreases Cellular ATP in Differentiated RGC-5 Cells. Investig. Ophthalmol. Vis. Sci..

[106] Coughlin L., Morrison R.S., Horner P.J., Inman D.M. (2015). Mitochondrial Morphology Differences and Mitophagy Deficit in Murine Glaucomatous Optic Nerve. Investig. Ophthalmol. Vis. Sci..

[107] Harun-Or-Rashid M., Pappenhagen N., Zubricky R., Coughlin L., Jassim A.H., Inman D.M. (2020). MCT2 overexpression rescues metabolic vulnerability and protects retinal ganglion cells in two models of glaucoma. Neurobiol. Dis..

[108] Lee S., Sheck L., Crowston J.G., Van Bergen N.J., O’Neill E.C., O’Hare F., Kong Y.X.G., Chrysostomou V., Vincent A.L., Trounce I. (2012). Impaired Complex-I-Linked Respiration and ATP Synthesis in Primary Open-Angle Glaucoma Patient Lymphoblasts. Investig. Ophthalmol. Vis. Sci..

[109] Van Bergen N.J., Crowston J.G., Craig J., Burdon K.P., Kearns L.S., Sharma S., Hewitt A.W., Mackey D.A., Trounce I.A. (2015). Measurement of Systemic Mitochondrial Function in Advanced Primary Open-Angle Glaucoma and Leber Hereditary Optic Neuropathy. PLoS ONE.

[110] Lascaratos G., Chau K., Zhu H., Gkotsi D., King R., Gout I., Kamal D., Luthert P., Schapira A., Garway-Heath D. (2015). Resistance to the most common optic neuropathy is associated with systemic mitochondrial efficiency. Neurobiol. Dis..

[111] Charliat G., Jolly D., Blanchard F. (1994). Genetic risk factor in primary open-angle glaucoma: A case-control study. Ophthalmic Epidemiol..

[112] Lascaratos G., Garway-Heath D., Willoughby C.E., Chau K., Schapira A. (2012). Mitochondrial dysfunction in glaucoma: Understanding genetic influences. Mitochondrion.

[113] Navarro A., Boveris A. (2007). The mitochondrial energy transduction system and the aging process. Am. J. Physiol. Physiol..

[114] Fraenkl S.A., Muser J., Groell R., Reinhard G., Orgül S., Flammer J., Goldblum D. (2011). Plasma Citrate Levels as a Potential Biomarker for Glaucoma. J. Ocul. Pharmacol. Ther..

[115] Field M.G., Yang D., Bian Z.-M., Petty H.R., Elner V.M. (2011). Retinal flavoprotein fluorescence correlates with mitochondrial stress, apoptosis, and chemokine expression. Exp. Eye Res..

[116] Glossmann H.H., Lutz O.M. (2019). Metformin and Aging: A Review. Gerontology.

[117] Li Y., Ryu C., Munie M., Noorulla S., Rana S., Edwards P., Gao H., Qiao X. (2018). Association of Metformin Treatment with Reduced Severity of Diabetic Retinopathy in Type 2 Diabetic Patients. J. Diabetes Res..

[118] Chen Y.-Y., Shen Y.-C., Lai Y.-J., Wang C.-Y., Lin K.-H., Feng S.-C., Liang C.-Y., Wei L.-C., Chou P. (2019). Association between Metformin and a Lower Risk of Age-Related Macular Degeneration in Patients with Type 2 Diabetes. J. Ophthalmol..

[119] Blitzer A.L., Ham S.A., Colby K.A., Skondra D. (2021). Association of Metformin Use with Age-Related Macular Degeneration. JAMA Ophthalmol..

[120] Anisimov V.N. (2013). Metformin: Do we finally have an anti-aging drug?. Cell Cycle.

[121] Vergroesen J., Stricker B., Ikram A., Kavousi M., Hofman A., Klaver C., Ramdas W. (2020). Systemic metformin use reduces open-angle glaucoma risk. Investig. Ophthalmol. Vis. Sci..

[122] Brown E., Ball J.D., Chen Z., Khurshid G.S., Prosperi M., Ash J.D. (2019). The Common Antidiabetic Drug Metformin Reduces Odds of Developing Age-Related Macular Degeneration. Investig. Ophthalmol. Vis. Sci..

[123] Meng S., Cao J., He Q., Xiong L., Chang E., Radovick S., Wondisford F.E., He L. (2015). Metformin Activates AMP-activated Protein Kinase by Promoting Formation of the αβγ Heterotrimeric Complex. J. Biol. Chem..

[124] Cufí S., Vazquez-Martin A., Oliveras-Ferraros C., Martín-Castillo B., Joven J., Menendez J. (2010). Metformin against TGFβ-induced epithelial-to-mesenchymal transition (EMT): From cancer stem cells to aging-associated fibrosis. Cell Cycle.

[125] Lin H., Li N., He H., Ying Y., Sunkara S., Luo L., Lv N., Huang D., Luo Z. (2015). AMPK Inhibits the Stimulatory Effects of TGF-β on Smad2/3 Activity, Cell Migration, and Epithelial-to-Mesenchymal Transition. Mol. Pharmacol..

[126] Li L., Huang W., Li K., Zhang K., Lin C., Han R., Lu C., Wang Y., Chen H., Sun F. (2015). Metformin attenuates gefitinib-induced exacerbation of pulmonary fibrosis by inhibition of TGF-β signaling pathway. Oncotarget.

[127] Lu J., Shi J., Li M., Gui B., Fu R., Yao G., Duan Z., Lv Z., Yang Y., Chen Z. (2015). Activation of AMPK by metformin inhibits TGF-β-induced collagen production in mouse renal fibroblasts. Life Sci..

[128] Kheirollahi V., Wasnick R.M., Biasin V., Vazquez-Armendariz A.I., Chu X., Moiseenko A., Weiss A., Wilhelm J., Zhang J.-S., Kwapiszewska G. (2019). Metformin induces lipogenic differentiation in myofibroblasts to reverse lung fibrosis. Nat. Commun..

[129] Thakur S., Viswanadhapalli S., Kopp J.B., Shi Q., Barnes J.L., Block K., Gorin Y., Abboud H.E. (2015). Activation of AMP-Activated Protein Kinase Prevents TGF-β1–Induced Epithelial-Mesenchymal Transition and Myofibroblast Activation. Am. J. Pathol..

[130] Dengler F. (2020). Activation of AMPK under Hypoxia: Many Roads Leading to Rome. Int. J. Mol. Sci..

[131] Ke R., Xu Q., Li C., Luo L., Huang D. (2018). Mechanisms of AMPK in the maintenance of ATP balance during energy metabolism. Cell Biol. Int..

[132] Shackelford D.B., Shaw R.J. (2009). The LKB1–AMPK pathway: Metabolism and growth control in tumour suppression. Nat. Rev. Cancer.

[133] Fujiwara Y., Kawaguchi Y., Fujimoto T., Kanayama N., Magari M., Tokumitsu H. (2016). Differential AMP-activated Protein Kinase (AMPK) Recognition Mechanism of Ca2+/Calmodulin-dependent Protein Kinase Kinase Isoforms. J. Biol. Chem..

[134] Lally J.S., Ghoshal S., DePeralta D.K., Moaven O., Wei L., Masia R., Erstad D.J., Fujiwara N., Leong V., Houde V.P. (2018). Inhibition of Acetyl-CoA Carboxylase by Phosphorylation or the Inhibitor ND-654 Suppresses Lipogenesis and Hepatocellular Carcinoma. Cell Metab..

[135] Marsin A.-S., Bertrand L., Rider M., Deprez J., Beauloye C., Vincent‡ M., Berghe‡ G.V.D., Carling D., Hue L. (2000). Phosphorylation and activation of heart PFK-2 by AMPK has a role in the stimulation of glycolysis during ischaemia. Curr. Biol..

[136] Gwinn D.M., Shackelford D.B., Egan D.F., Mihaylova M.M., Mery A., Vasquez D.S., Turk B.E., Shaw R.J. (2008). AMPK Phosphorylation of Raptor Mediates a Metabolic Checkpoint. Mol. Cell.

[137] Chatterjee A., Villarreal G., Oh D.-J., Kang M.H., Rhee D.J. (2014). AMP-Activated Protein Kinase Regulates Intraocular Pressure, Extracellular Matrix, and Cytoskeleton in Trabecular Meshwork. Investig. Ophthalmol. Vis. Sci..

[138] Belforte N., Vargas J.L.C., Di Polo A. (2018). AB015. Metabolic stress in glaucoma engages early activation of the energy biosensor adenosine monophosphate-activated protein kinase leading to neuronal dysfunction. Ann. Eye Sci..

[139] Harun-Or-Rashid M., Inman D.M. (2018). Reduced AMPK activation and increased HCAR activation drive anti-inflammatory response and neuroprotection in glaucoma. J. Neuroinflamm..

[140] Xiao H., Zhang J., Xu Z., Feng Y., Zhang M., Liu J., Chen R., Shen J., Wu J., Lu Z. (2016). Metformin is a novel suppressor for transforming growth factor (TGF)-β1. Sci. Rep..

[141] Kalender A., Selvaraj A., Kim S.Y., Gulati P., Brûlé S., Viollet B., Kemp B., Bardeesy N., Dennis P., Schlager J.J. (2010). Metformin, Independent of AMPK, Inhibits mTORC1 in a Rag GTPase-Dependent Manner. Cell Metab..

[142] Obara A., Fujita Y., Abudukadier A., Fukushima T., Oguri Y., Ogura M., Harashima S.-I., Hosokawa M., Inagaki N. (2015). DEPTOR-related mTOR suppression is involved in metformin’s anti-cancer action in human liver cancer cells. Biochem. Biophys. Res. Commun..

[143] Zheng W., Song J., Zhang Y., Chen S., Ruan H., Fan C. (2017). Metformin prevents peritendinous fibrosis by inhibiting transforming growth factor-β signaling. Oncotarget.

[144] Sato N., Takasaka N., Yoshida M., Tsubouchi K., Minagawa S., Araya J., Saito N., Fujita Y., Kurita Y., Kobayashi K. (2016). Metformin attenuates lung fibrosis development via NOX4 suppression. Respir. Res..

[145] Shanmugam G., Narasimhan M., Tamowski S., Darley-Usmar V., Rajasekaran N.S. (2017). Constitutive activation of Nrf2 induces a stable reductive state in the mouse myocardium. Redox Biol..

[146] Li X., Leng Y., Jiang Q., Wang Z., Luo P., Zhang C., Chen L., Wang Y., Wang H., Yue X. (2020). Eye Drops of Metformin Prevents Fibrosis After Glaucoma Filtration Surgery in Rats via Activating AMPK/Nrf2 Signaling Pathway. Front. Pharmacol..

[147] Du J., Zhu M., Li H., Liang G., Li Y., Feng S. (2020). Metformin attenuates cardiac remodeling in mice through the Nrf2/Keap1 signaling pathway. Exp. Ther. Med..

[148] Rangarajan S., Bone N.B., Zmijewska A.A., Jiang S., Park D.W., Bernard K., Locy M.L., Ravi S., Deshane J., Mannon R.B. (2018). Author Correction: Metformin reverses established lung fibrosis in a bleomycin model. Nat. Med..

[149] Cavaglieri R.C., Day R.T., Feliers D., Abboud H.E. (2015). Metformin prevents renal interstitial fibrosis in mice with unilateral ureteral obstruction. Mol. Cell. Endocrinol..

[150] Kim H., Moon S.Y., Kim J.-S., Baek C.H., Kim M., Min J.Y., Lee S.K. (2015). Activation of AMP-activated protein kinase inhibits ER stress and renal fibrosis. Am. J. Physiol. Physiol..

[151] Wang Y., Wang Y., Li Y., Lu L., Peng Y., Zhang S., Xia A. (2020). Metformin attenuates renal interstitial fibrosis through upregulation of Deptor in unilateral ureteral obstruction in rats. Exp. Ther. Med..

[152] Mummidi S., Das N.A., Carpenter A.J., Kandikattu H., Krenz M., Siebenlist U., Valente A.J., Chandrasekar B. (2016). Metformin inhibits aldosterone-induced cardiac fibroblast activation, migration and proliferation in vitro, and reverses aldosterone+salt-induced cardiac fibrosis in vivo. J. Mol. Cell. Cardiol..

[153] Xiao H., Ma X., Feng W., Fu Y., Lu Z., Xu M., Shen Q., Zhu Y., Zhang Y. (2010). Metformin attenuates cardiac fibrosis by inhibiting the TGFβ1–Smad3 signalling pathway. Cardiovasc. Res..

[154] Fontaine E. (2018). Metformin-Induced Mitochondrial Complex I Inhibition: Facts, Uncertainties, and Consequences. Front. Endocrinol..

[155] Hou W.-L., Yin J., Alimujiang M., Yu X.-Y., Ai L.-G., Bao Y.-Q., Liu F., Jia W.-P. (2017). Inhibition of mitochondrial complex I improves glucose metabolism independently of AMPK activation. J. Cell. Mol. Med..

[156] Chauvin C., De Oliveira F., Ronot X., Mousseau M., Leverve X., Fontaine E. (2001). Rotenone Inhibits the Mitochondrial Permeability Transition-induced Cell Death in U937 and KB Cells. J. Biol. Chem..

[157] Owen M.R., Doran E., Halestrap A.P. (2000). Evidence that metformin exerts its anti-diabetic effects through inhibition of complex 1 of the mitochondrial respiratory chain. Biochem. J..

[158] Bridges H.R., Jones A.J.Y., Pollak M.N., Hirst J. (2014). Effects of metformin and other biguanides on oxidative phosphorylation in mitochondria. Biochem. J..

[159] Wheaton W.W., Weinberg S., Hamanaka R.B., Soberanes S., Sullivan L., Anso E., Glasauer A., Dufour E., Mutlu G.M., Budigner G.S. (2014). Metformin inhibits mitochondrial complex I of cancer cells to reduce tumorigenesis. eLife.

[160] El-Mir M.-Y., Nogueira V., Fontaine E., Avéret N., Rigoulet M., Leverve X. (2000). Dimethylbiguanide Inhibits Cell Respiration via an Indirect Effect Targeted on the Respiratory Chain Complex I. J. Biol. Chem..

[161] Vial G., Detaille D., Guigas B. (2019). Role of Mitochondria in the Mechanism(s) of Action of Metformin. Front. Endocrinol..

[162] Bailey C.J. (2017). Metformin: Historical overview. Diabetologia.

[163] Foretz M., Guigas B., Bertrand L., Pollak M., Viollet B. (2014). Metformin: From Mechanisms of Action to Therapies. Cell Metab..

[164] Petronilli V., Penzo D., Scorrano L., Bernardi P., Di Lisa F. (2001). The Mitochondrial Permeability Transition, Release of Cytochrome c and Cell Death. J. Biol. Chem..

[165] Fontaine E., Eriksson O., Ichas F., Bernardi P. (1998). Regulation of the Permeability Transition Pore in Skeletal Muscle Mitochondria. J. Biol. Chem..

[166] Batandier C., Leverve X., Fontaine E. (2004). Opening of the Mitochondrial Permeability Transition Pore Induces Reactive Oxygen Species Production at the Level of the Respiratory Chain Complex I. J. Biol. Chem..

[167] De Giorgi F., Lartigue L., Bauer M.K.A., Schubert A., Grimm S., Hanson G.T., Remington S.J., Youle R.J., Ichas F. (2002). The permeability transition pore signals apoptosis by directing Bax translocation and multimerization. FASEB J..

[168] Detaille D., Guigas B., Chauvin C., Batandier C., Fontaine E., Wiernsperger N., Leverve X. (2005). Metformin Prevents High-Glucose-Induced Endothelial Cell Death Through a Mitochondrial Permeability Transition-Dependent Process. Diabetes.

[169] Guigas B., Detaille D., Chauvin C., Batandier C., De Oliveira F., Fontaine E., Leverve X. (2004). Metformin inhibits mitochondrial permeability transition and cell death: A pharmacological in vitro study. Biochem. J..

[170] Boukalova S., Stursa J., Werner L., Ezrová Z., Černý J., Bezawork-Geleta A., Pecinova A., Dong L., Drahota Z., Neuzil J. (2016). Mitochondrial Targeting of Metformin Enhances Its Activity against Pancreatic Cancer. Mol. Cancer Ther..

[171] Wu Y., Gao W.-N., Xue Y.-N., Zhang L.-C., Zhang J.-J., Lu S.-Y., Yan X.-Y., Yu H.-M., Su J., Sun L.-K. (2018). SIRT3 aggravates metformin-induced energy stress and apoptosis in ovarian cancer cells. Exp. Cell Res..

[172] Wang Y., An H., Liu T., Qin C., Sesaki H., Guo S., Radovick S., Hussain M., Maheshwari A., Wondisford F.E. (2019). Metformin Improves Mitochondrial Respiratory Activity through Activation of AMPK. Cell Rep..

[173] Yang M., Darwish T., Larraufie P., Rimmington D., Cimino I., Goldspink D.A., Jenkins B., Koulman A., Brighton C.A., Ma M. (2021). Inhibition of mitochondrial function by metformin increases glucose uptake, glycolysis and GDF-15 release from intestinal cells. Sci. Rep..

[174] Ishihara N., Fujita Y., Oka T., Mihara K. (2006). Regulation of mitochondrial morphology through proteolytic cleavage of OPA1. EMBO J..

[175] Larsen S., Rabøl R., Hansen C.N., Madsbad S., Helge J., Dela F. (2011). Metformin-treated patients with type 2 diabetes have normal mitochondrial complex I respiration. Diabetologia.

[176] Victor V.M., Rovira-Llopis S., Bañuls C., Diaz-Morales N., Castelló R., Falcón R., Gómez M., Rocha M., Hernández-Mijares A. (2015). Effects of metformin on mitochondrial function of leukocytes from polycystic ovary syndrome patients with insulin resistance. Eur. J. Endocrinol..

[177] Dowling R.J., Lam S., Bassi C., Mouaaz S., Aman A., Kiyota T., Al-Awar R., Goodwin P., Stambolic V. (2016). Metformin Pharmacokinetics in Mouse Tumors: Implications for Human Therapy. Cell Metab..

[178] Iversen A.B., Horsman M., Jakobsen S., Jensen J.B., Garm C., Jessen N., Breining P., Frøkiær J., Busk M. (2017). Results from 11C-metformin-PET scans, tissue analysis and cellular drug-sensitivity assays questions the view that biguanides affects tumor respiration directly. Sci. Rep..

[179] Alshawi A., Agius L. (2019). Low metformin causes a more oxidized mitochondrial NADH/NAD redox state in hepatocytes and inhibits gluconeogenesis by a redox-independent mechanism. J. Biol. Chem..

[180] Cameron A.R., Logie L., Patel K.A., Erhardt S., Bacon S., Middleton P., Harthill J., Forteath C., Coats J.T., Kerr C. (2017). Metformin selectively targets redox control of complex I energy transduction. Redox Biol..

[181] Williams P.A., Harder J.M., Foxworth N.E., Cochran K.E., Philip V.M., Porciatti V., Smithies O., John S.W.M. (2017). Vitamin B3modulates mitochondrial vulnerability and prevents glaucoma in aged mice. Science.

[182] Lau C. (2009). The NMN/NaMN adenylyltransferase (NMNAT) protein family. Front. Biosci..

[183] Di Lisa F., Ziegler M. (2001). Pathophysiological relevance of mitochondria in NAD+metabolism. FEBS Lett..

[184] Williams P.A., Harder J.M., Cardozo B.H., Foxworth N.E., John S.W.M. (2018). Nicotinamide treatment robustly protects from inherited mouse glaucoma. Commun. Integr. Biol..

[185] Cimaglia G., Votruba M., Morgan J.E., André H., Williams P.A. (2020). Potential Therapeutic Benefit of NAD^+^ Supplementation for Glaucoma and Age-Related Macular Degeneration. Nutrients.

[186] Tribble J.R., Otmani A., Sun S., Ellis S.A., Cimaglia G., Vohra R., Jöe M., Lardner E., Venkataraman A.P., Domínguez-Vicent A. (2021). Nicotinamide provides neuroprotection in glaucoma by protecting against mitochondrial and metabolic dysfunction. Redox Biol..

[187] Rao U.S. (2010). Diagnosing, Preventing, and Treating Glaucoma. AMA J. Ethics.

[188] Ahmad S.S. (2018). Glaucoma suspects: A practical approach. Taiwan J. Ophthalmol..

[189] Zou T., He J., Chen X., Sun D., Fan X., Xu H. (2019). Rescue of Retinal Degeneration in rd1 Mice by Intravitreally Injected Metformin. Front. Mol. Neurosci..

[190] Zhongshan Ophthalmic Center, Sun Yat-Sen University (2019). Effect of Metformin on Visual Function in Patients with Glaucoma.

